# Idiopathic Granulomatous Mastitis-A Mystery Yet to be Unraveled: A Case Series and Review of Literature

**DOI:** 10.7759/cureus.6895

**Published:** 2020-02-05

**Authors:** Vinay Mathew Thomas, Swetha Ann Alexander, Poorva Bindal, James Vredenburgh

**Affiliations:** 1 Internal Medicine, University of Connecticut , Farmington, USA; 2 Internal Medicine, University of Connecticut, Farmington, USA; 3 Hematology and Oncology, Beth Israel Deaconess Medical Center, Boston, USA; 4 Hematology and Oncology, Saint Francis Hospital and Medical Center, Hartford, USA

**Keywords:** idiopathic granulomatous mastitis, autoimmunity, breast cancer, breast mass, inflammatory breast disease

## Abstract

Idiopathic granulomatous mastitis (IGM) is a chronic inflammatory disease of the breast, the etiology of which, has still not been elucidated. There have been several mechanisms proposed to explain the pathogenesis. Since the first description of the disease, it has proved itself to be a great diagnostic and therapeutic challenge. It is very often misdiagnosed as cancer, resulting in myriad workup by the physician and great distress to the patient. Clear guidelines as to the management have still not been described. Here, we describe two patients who presented with IGM and have been successfully treated. The first patient was treated with a combination of steroids and antibiotics. The second patient achieved remission of the disease with antibiotics alone. We also propose an algorithm for the management of IGM.

## Introduction

Idiopathic granulomatous mastitis (IGM) is a chronic inflammatory disease of the breast of unclear etiology [1]. Since the first report of the disease in 1972 by Kessler and Wolloch, IGM has posed great diagnostic and therapeutic dilemmas [2]. An incidence of 2.4 cases per 100,000 women aged 20-40 years has been reported in the US, with increased frequency among Hispanic women [3-4]. IGM is most commonly seen in women of childbearing age, although there have been reports in men and elderly women [5]. Several mechanisms have been proposed to explain the pathogenesis of IGM that includes pregnancy, lactation, oral contraceptive pill (OCP) use, hyperprolactinemia, and autoimmunity. Autoimmunity seems to be a likely mechanism, based on the response of IGM to treatment with steroids and the histological findings of T-lymphocyte predominance [1]. IGM can present as a palpable mass, abscess formation, nipple retraction, peau d’ orange or axillary lymphadenopathy [6]. The similarity to the clinical presentation of breast cancer often leads to difficulty in diagnosis, resulting in misdiagnosis as cancer, delayed diagnosis and misguided therapies [1,7]. Imaging modalities are often inconclusive and core needle biopsy seems to be the most accurate way to diagnose IGM [8]. There has been no consensus on how to treat IGM and currently, no guidelines exist as to what the best algorithm of treatment would be. Steroids have become the mainstay of treatment in recent years. Other approaches to treatment include observation, antibiotics, and surgery.

We present a case series of two cases of IGM, which were successfully treated.

## Case presentation

Patient 1

**Figure 1:**
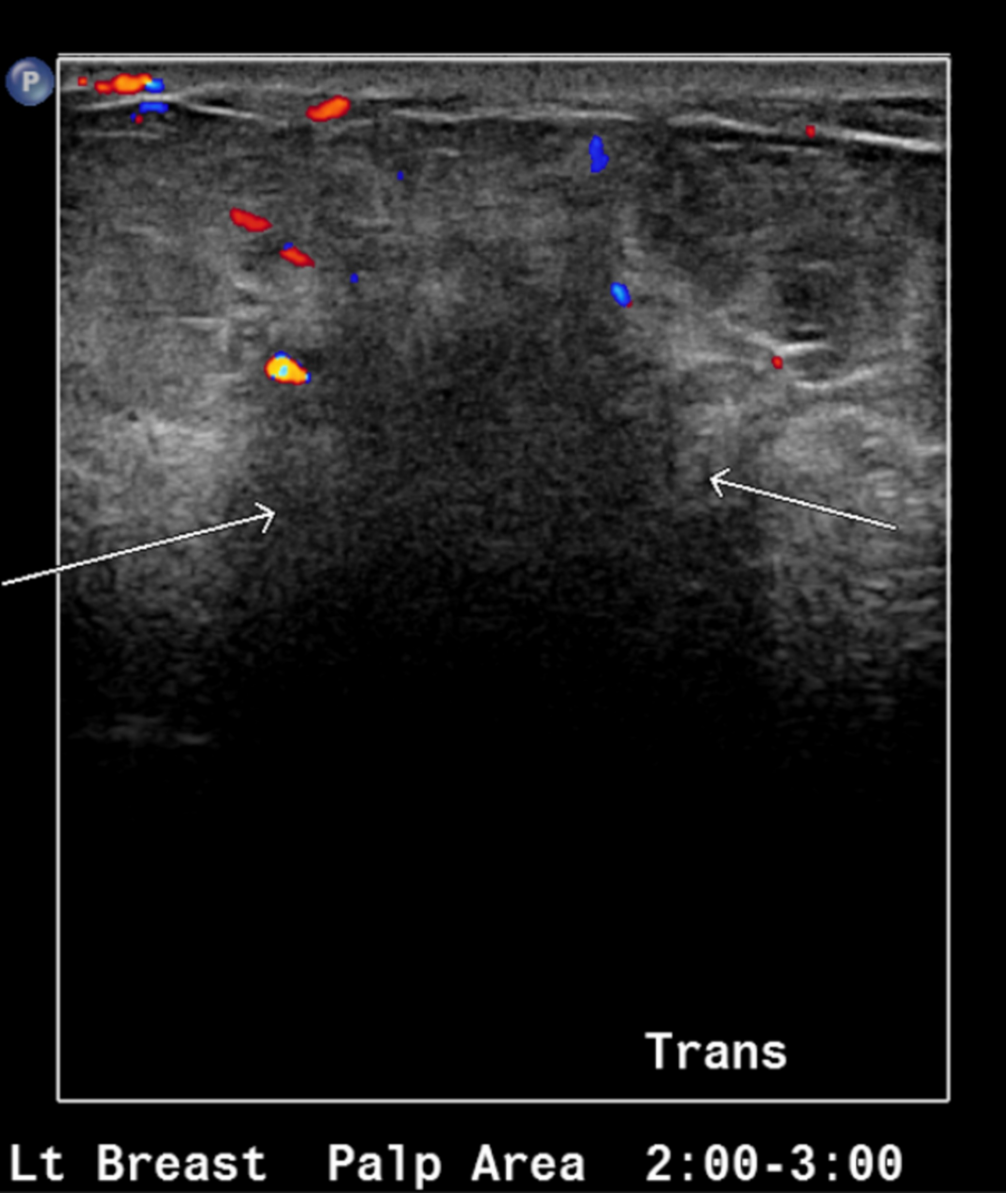
Ultrasound of the left breast showing area of abnormal echogenicity

**Figure 2:**
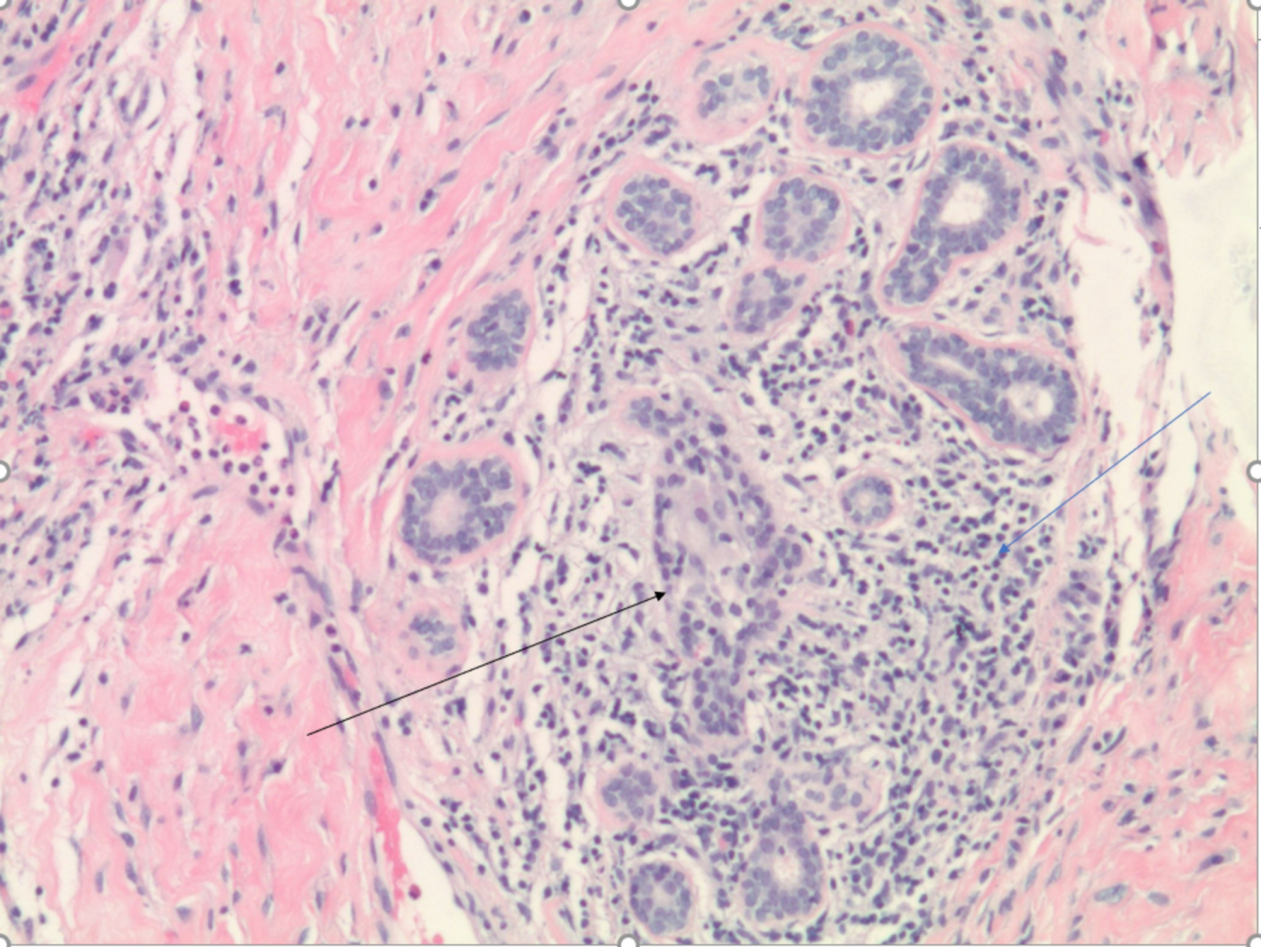
Fine-needle aspiration showing breast tubules with lymphocytic infiltration (blue arrow) and granulomas (black arrow)

A 46-year-old woman presented with a left breast mass and associated breast pain. There was also a 1.4 cm lymph node within the left axilla. She underwent bilateral diagnostic mammography, which showed increased density in the left breast. She subsequently underwent a bilateral breast ultrasound, which revealed an ill-defined area of abnormal echogenicity in the left breast (Figure 1). No mass or collection was seen on ultrasound. A fine-needle aspiration (FNA) was performed, which showed fibrofatty breast tissue with acute and chronic mastitis, with evidence of lymphocytic infiltration and non-caseating granulomas (Figure 2). No evidence of malignancy was seen. She was initially started on a long course of antibiotics, which initially resulted in some improvement of symptoms. She then developed painful erythematous nodules and joint pain in ankles and feet. She was then started on oral prednisone after her symptoms worsened. Pus from her breast drainage was cultured and grew *Corynebacterium kroppenstedtii.* She was started on a course of doxycycline and trimethoprim-sulfamethoxazole. The steroids resulted in significant improvement of the disease, with the resolution of pain, erythema, and abscess formation. Her steroid dose has been slowly tapered but has been unable to come off steroids over the past two years due to disease recurrence

Patient 2

**Figure 3:**
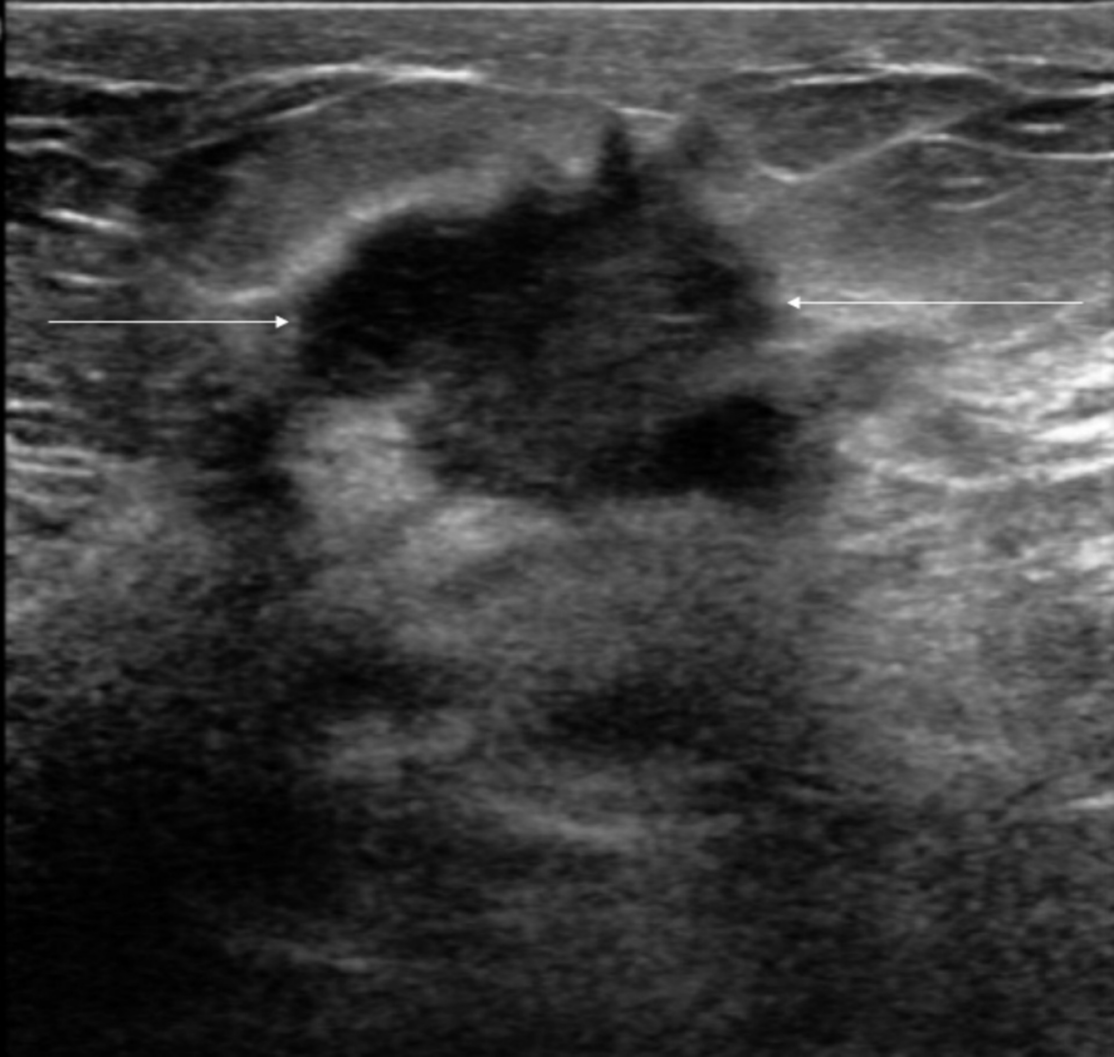
Ultrasound of the right breast showing the hypoechoic region

**Figure 4:**
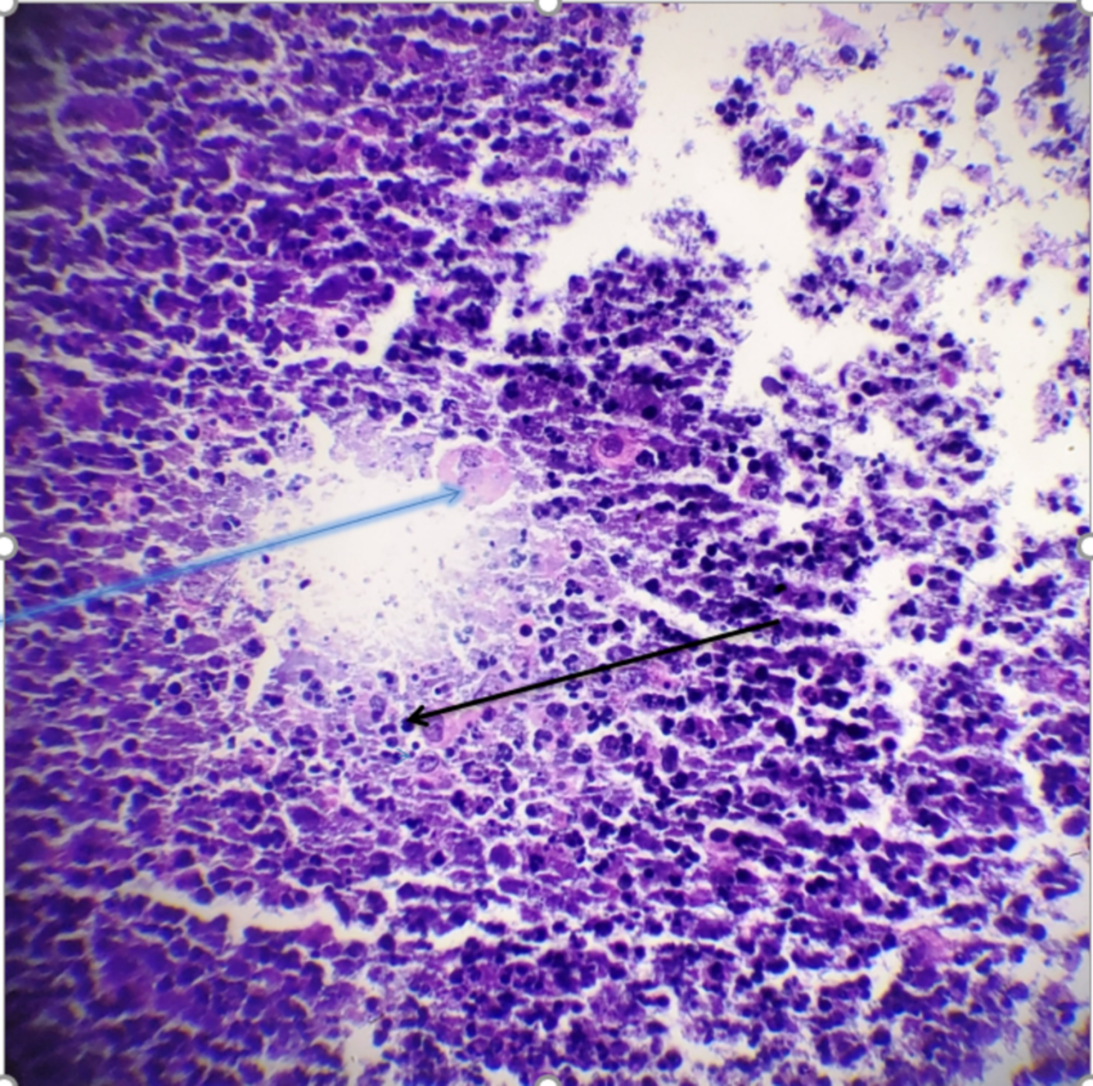
Fine-needle aspiration showing histiocytes (blue arrow) and inflammatory cells (black arrow)

The second patient is a 30-year-old female, who initially presented with a right breast mass and erythema. She then underwent a mammogram, which revealed a possible mass in the right breast as well as left. Following this, she underwent an ultrasound, which showed no obvious lesion in the left breast but showed a hypoechoic region in the right breast (Figure 3). An FNA was done and cytology showed findings suggestive of granulomatous mastitis, with histiocytes and other inflammatory cells (Figure 4). She was initially started on trimethoprim-sulfamethoxazole with no improvement symptoms. She developed new abscesses that were subsequently drained. She was then placed on doxycycline with significant improvement of symptoms and has been able to avoid steroids.

## Discussion

 IGM is a rare, chronic inflammatory condition that can mimic malignancy and can often be difficult to diagnose and manage. Since the first report of the case in 1972, there have been several reports of the condition, but clear-cut management of the disease has still not emerged. As the name suggests, the etiology of IGM has been difficult to define. Three leading hypotheses for possible etiologies are: autoimmune, infectious, and hormonal [9]. Of these, autoimmunity has been accepted as the most likely cause [9-11]. The hypothesis that IGM might be an autoimmune condition has emerged from the fact that there is a T-lymphocytic predominance in IGM evidenced by histochemical studies and also because several studies have shown an excellent response of IGM to steroids [8-9]. It has been postulated that epithelial damage to the mammary ducts, which in turn results in extravasation of milk protein within the interstitial tissues of the breast triggers the autoimmune reaction [1]. The damage to the lobules of the breast can, in turn, be caused by trauma to the breasts, chemical irritation [12]. Infection can also trigger autoimmunity by damage to the breast lobules. Altintoprak et al. tried to find objective evidence for the autoimmune basis of the disease by measuring the anti-nuclear antibody (ANA) and extractable nuclear antigen (ENA) levels of patients diagnosed with IGM [13]. However, they were unable to demonstrate any levels which could support the etiology as autoimmunity. With regard to the infectious etiology, it has been proposed that infections have more of an association than a causal relation with IGM. Taylor et al. demonstrated the presence of *Corynebacterium* in 34 cases of women with granulomatous mastitis [14]. *Corynebacterium* is a gram-positive bacterium and is usually present as a normal commensal on the skin. *Corynebacterium* has frequently been implicated in IGM and is the most common bacterial strain found in cases of IGM [15]. *Corynebacterium kroppenstedtii*, *Corynebacterium amycolatum,* and *Corynebacterium tuberculostearicum* have been isolated in decreasing frequency in cases of IGM [1]. In the first patient in our report, *Corynebacterium kroppenstedtii* was isolated. The hormonal etiology has not been proven either. Although IGM does occur more in women of childbearing age, with a peak incidence in pregnant and lactating women, there have been reports of IGM occurring in nulliparous women or even several years after the last pregnancy [16]. Several studies have suggested the role of OCPs in IGM, but no definite relationship has been identified [2,12]. High prolactin levels have been reported to have an association with IGM. However, the cases of IGM with reported high prolactin levels have been those that were either drug-induced or recurrent disease [10]. No causal relationship has been established in past studies and thus the relation of IGM with hormonal status remains unclear. Our patients had normal prolactin levels.

The spectrum of presentation of IGM is extremely wide. The most common presentation is a unilateral mass that can occur in any quadrant of the breast [3,9,17,18]. The upper outer quadrant can be the most commonly involved site [1,18]. The mass may be painful and can be associated with erythema of the overlying skin. Often, peau d’ orange appearance of the skin and nipple retraction may also be seen, which makes it very similar in appearance to malignancies [1]. It can also present with abscesses and draining tracts or fistulae [18]. Extra-mammary presentations include axillary lymphadenopathy, erythema nodosum, and arthritis [9].

The diagnosis of IGM requires a high index of suspicion after other causes of granulomatous mastitis have been excluded. These include causes like mycobacterial infections, sarcoidosis, foreign body reactions, and fat necrosis. Tuberculous mastitis must be ruled out because steroids are the mainstay of treatment of IGM, which can aggravate mycobacterial infections. All specimens must undergo Ziehl acid-fast staining [1]. 

There are no specific radiological findings that could point towards a diagnosis of IGM. The workup for IGM can be done in the same way as a breast mass would be evaluated, i.e., with a mammogram in a woman above 30 years of age and with ultrasound in a woman below 30 years of age. There are no pathognomonic mammographic findings that would point towards a diagnosis. The most common mammographic findings include focal areas of asymmetry, which can be single or multiple. Skin thickening and axillary lymphadenopathy can also be seen [3,11]. The most common sonographic finding is that of a heterogeneously echogenic irregularly shaped mass with no distinct margins. The tubular configuration is seen in a majority of cases [1,3]. Magnetic resonance imaging (MRI) lacks the ability to distinguish between a tumor and an inflammatory process. Thus, imaging studies cannot be used to make a definite diagnosis, and thus tissue diagnosis is employed for the same.

A definitive diagnosis of IGM is based on histopathological examination of the tissue specimen. FNA has been historically used but is now losing favor in the diagnosis of IGM due to its lower sensitivity. Studies have shown that the diagnostic yield of FNA may be as low as 21%-39% [1]. FNA may also not differentiate between IGM and other granulomatous conditions of the breast [11]. For a definite diagnosis, FNA must often be followed by a core biopsy. This subjects patients to unnecessary testing. Core needle biopsy has a higher diagnostic yield and often has sensitivity upwards of 94% based on studies [11]. In some cases, where core needle biopsy is not diagnostic, an open biopsy can be considered [1,11]. However, open biopsy is controversial because it often leads to scarring and sometimes non-healing ulcers or sinus tracts [11]. IGM is characterized by the presence of non-caseating granulomas centered around breast lobules, which are composed of histiocytes and Langhans giant cells. There is also a chronic inflammatory picture with lymphocytes and plasma cells interspersed with microabscesses [1].

Although there have been several reports of IGM, there has been no universally accepted algorithm for the treatment of IGM. The treatment options, which have been tried include observation, steroids, antibiotics, immunosuppressive agents like methotrexate and surgery. Observation can be sometimes used as an initial approach in patients who present with an initial episode of IGM. This observation is based on studies that reported successful management with conservative management. Lai et al. in their study reported spontaneous resolution of IGM in 50% of cases and stable disease in the other half [17]. However, the issue with the observational method is that the resolution can sometimes take a long period of time, as seen in the study by Lai and colleagues (approximately two years). This may not be appropriate in the clinical setting. Also, observation may not be appropriate in patients with symptomatic disease.

Antibiotics have also been used in treatment. Although, this is controversial as no direct causation has been established between bacterial infiltration and IGM. Antibiotic use can be guided by microbiological data available, with chosen agents being directed against gram-positive organisms [1]. In our patients, therapy was directed against Corynebacterium with mild improvement in symptoms. Antibiotics can also be used in conjunction with incision and drainage, especially where abscesses have formed. 

At this time, corticosteroids are the mainstay of treatment for IGM. Several studies have shown favorable results with steroid use. In a prospective study by Pandey et al., of the 44 patients treated with steroids, there was complete resolution of disease in 35 patients (79.5%) [8]. The median time to resolution was 5.3 months. A total of 23% had a recurrence of the disease and all of them resolved with the second course of steroids [8]. In a retrospective study done by Oran et al., 25 out of 46 patients with IGM were treated with steroids. Of these 25 patients, only three (7%) failed to respond to steroids and required surgical excision [16]. Other studies have demonstrated a similar benefit with steroid therapy. The most commonly recommended duration of steroid treatment is three to six months. Doses as high as 60 mg of prednisone daily can be used with a gradual taper [1]. However, long-term steroid therapy leads to several side effects, which include weight gain, hypertension, glucose intolerance, Cushing’s syndrome, and steroid myopathy. Immunosuppressive agents like methotrexate and azathioprine can also be used in cases that are resistant to steroid therapy and can function as steroid-sparing agents [19].

Before the introduction of steroids, IGM was managed primarily with surgery. In the retrospective study done by Oran et al., there were 46 patients being followed by the same surgical team. Wide local excision with negative margins was performed in 18 cases. Of these 18 cases, only three recurred who were then treated successfully with steroids and re-excision [16]. The authors emphasized the importance of wide local excision with negative margins to decrease the risk of recurrence. However, surgical excision has been falling out of favor because of the good response of IGM to steroid therapy and also the fact that surgical treatment is associated with a high rate of disfigurement, fistula formation, and poor wound healing [20].

**Figure 5:**
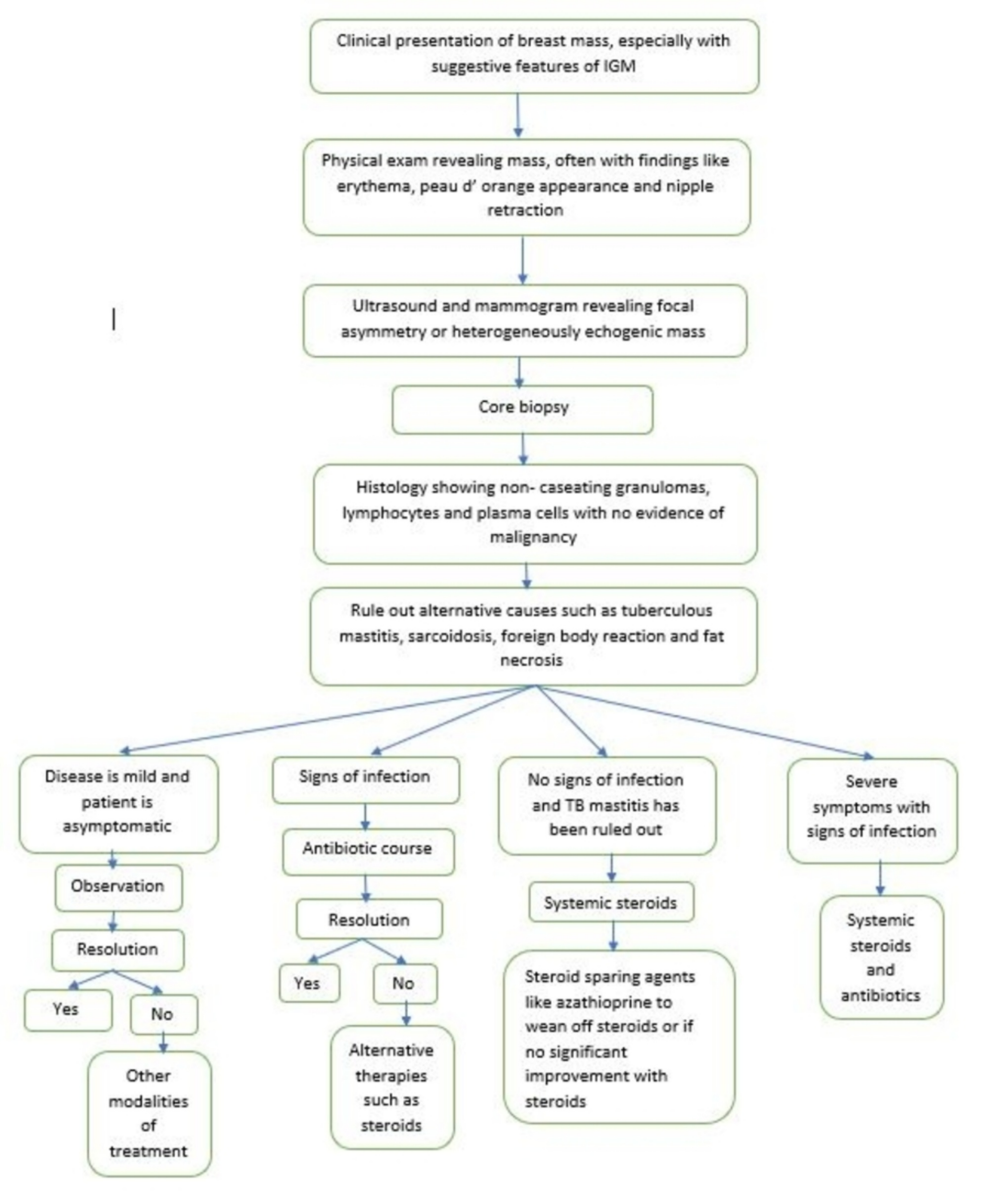
Proposed algorithm for the management of idiopathic granulomatous mastitis

Here, we propose an algorithm for the management of IGM (Figure 5).

## Conclusions

IGM is a chronic and often debilitating disease. It requires a very astute clinician to diagnose the condition. With no accepted guidelines for the treatment of the disease, it becomes extremely challenging to adequately treat the condition. Further larger studies need to be undertaken to elucidate the most appropriate way of managing this condition.
